# Increased risk of neurodevelopmental impairment associated with reduced brain volume at term-equivalent age in preterm infants with germinal matrix hemorrhage

**DOI:** 10.1007/s00234-026-03929-6

**Published:** 2026-03-03

**Authors:** Chanyoung Rhee, Young Hun Choi, Seung Han Shin, Seh Hyun Kim, Yeon Jin Cho, Seunghyun Lee, Jung-Eun Cheon, Jae-Yeon Hwang

**Affiliations:** 1 Department of Radiology, Seoul National University Hospital, Seoul, Korea, Republic of; 2 Department of Radiology, College of Medicine, Seoul National University, Seoul, Korea, Republic of; 3 Institute of Radiation Medicine, Seoul National University Medical Research Center, Seoul, Korea, Republic of; 4 Department of Pediatrics, Seoul National University Children’s Hospital, Seoul, Korea, Republic of; 5 Department of Pediatrics, College of Medicine, Seoul National University, Seoul, Korea, Republic of

**Keywords:** Germinal matrix hemorrhage, Neurodevelopmental impairment, Deep learning, Preterm infant, Punctate white matter lesion

## Abstract

**Purpose:**

To assess the association of low-grade germinal matrix–intraventricular hemorrhage (GM-IVH) with reduced supratentorial and white matter volumes at term-equivalent age, and to evaluate whether these volumes are related to neurodevelopmental impairment (NDI).

**Methods:**

This study included preterm infants (gestational age at birth < 37 weeks) who underwent cranial ultrasound within three days of birth and three-dimensional T1-weighted brain magnetic resonance imaging at 34–46 weeks’ postmenstrual age from December 2020 to August 2024. Participants were categorized into the control, GMH (Papile grades 1–2), and punctate white matter lesion (PWML) groups; infants with severe conditions were excluded. A U-Net-based algorithm was used to automatically segment supratentorial and white matter volumes. NDI was defined as a motor score ≤ 85 or cognitive and language scores both ≤ 85 on the Bayley-III. Standardized residuals were calculated to assess volume deviation from the sex-specific reference (control group) and compared according to low-grade GM-IVH and NDI status. A two-way analysis of variance with Tukey’s post hoc test was used for statistical comparison.

**Results:**

A total of 171 infants (89 girls; gestational age, 28.32 ± 2.76 weeks; postmenstrual age, 37.81 ± 2.36 weeks) were included, with 119 in the control group, 41 in the GMH group, and 11 in the PWML group. The supratentorial volume-PMA slope was lower in the GMH and PWML groups than in the control group (19.37 cm³/week for control; 12.37 cm³/week for GMH, *P* = 0.02; 11.49 cm³/week for PWML, *P* = 0.04). In the GMH group, significant volume differences were observed between the NDI and non-NDI subgroups (standardized residuals: -1.12 ± 0.58 vs. 0.35 ± 0.69 for supratentorial volume; -0.95 ± 0.68 vs. 0.29 ± 0.92 for white matter volume; both *P* < 0.001), whereas no significant differences were found in the control group.

**Conclusion:**

Low-grade GM-IVH with reduced supratentorial or white matter volume is associated with an increased risk of NDI in preterm infants.

## Introduction

 Germinal matrix–intraventricular hemorrhage (GM-IVH) is a common complication of prematurity that originates from bleeding in the fragile germinal matrix adjacent to the lateral ventricles [1, 2]. Its incidence is inversely related to gestational age at birth [3, 4]. GM-IVH is categorized as low-grade when hemorrhage is confined to the germinal matrix or ventricle without hydrocephalus, and as high-grade when hydrocephalus is present [5]. High-grade disease is a well-established risk factor for cognitive, language, and motor impairments, as well as cerebral palsy and sensory deficits [6–8]. In contrast, the long-term neurodevelopmental impact of low-grade GM-IVH remains controversial, with studies reporting conflicting associations with neurodevelopmental impairment (NDI) [9–12]. In addition to GM-IVH, punctate white-matter lesions (PWML) are frequently observed on neonatal magnetic resonance imaging (MRI) and are regarded as another form of mild brain injury in preterm infants, but their prognostic significance is still debated [13–15].

Several studies have shown that smaller brain volumes at term-equivalent age are associated with poorer cognitive, language, and motor outcomes at two years in preterm and congenital heart disease infants [16, 17]. Given that the germinal matrix is densely populated with neurogenic progenitors, a volume deficit following GM-IVH may reflect injury-related tissue loss. In this context, volume loss in GM-IVH may serve as a prognostic factor for NDI. Validating this hypothesis requires precise brain volumetry, which is now achievable through deep-learning segmentation across various age groups, including infants [18–20].

This study aimed to identify pathological brain volume reduction in preterm infants with mild brain injury, including low-grade GM-IVH and PWML. A U-Net-based model, previously validated against manual segmentation, was used to estimate supratentorial and white matter volumes [18]. Furthermore, we investigated whether reduced brain volume in these infants is associated with adverse neurodevelopmental outcomes.

## Methods

This retrospective study was approved by our Institutional Review Board (Seoul National University Hospital IRB No. H-2505-045-1637), and the requirement for patient informed consent was waived.

### Study cohort

**Fig. 1:**
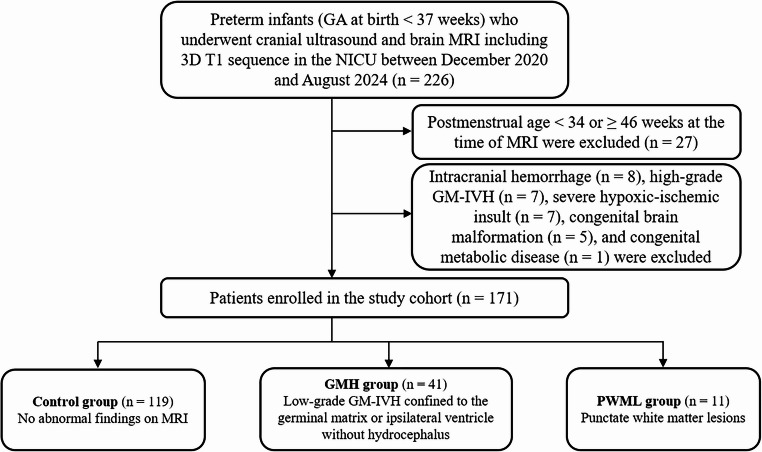
Flow diagram of the study. GA, gestational age; MRI, magnetic resonance imaging; 3D, three-dimensional; NICU, neonatal intensive care unit; GM-IVH, germinal matrix-intraventricular hemorrhage; PWML, punctate white matter lesion

This study enrolled preterm infants (gestational age at birth < 37 weeks) who underwent cranial ultrasound within three days of birth and brain MRI with three-dimensional (3D) T1-weighted imaging in the neonatal intensive care unit between December 2020 and August 2024. Among these, we included MRI scans performed between 34 and 46 weeks of postmenstrual age (PMA). Infants with high-grade GM-IVH (Papile grade III or IV), severe hypoxic–ischemic insult, intracranial hemorrhage, congenital brain malformations, or congenital metabolic diseases were excluded. Two radiologists (C.R. and J.Y.H., with 2 and 18 years of pediatric MRI interpretation experience, respectively) independently reviewed cranial ultrasound and MRI to categorize patients into control, GMH (low-grade GM-IVH, Papile grade I or II), and PWML groups. Low-grade GM–IVH, as defined by Papile grades I and II in this study, largely corresponds to Volpe categories without ventricular dilatation or progression [21]. PWML was identified as T1-hyperintense and T2-hypointense lesions in the cerebral white matter, with no cystic changes or blooming artifacts on gradient-recalled echo sequences [22]. Group assignments were made by consensus between the two radiologists in cases of disagreement. A flow diagram of the study is shown in Fig. 1.

For the assessment of NDI, patients who underwent the Bayley-III test at 45–51 months of age were selected from the cohort. NDI was defined as a motor score ≤ 85 or both cognitive and language scores ≤ 85 [23]. When patients had multiple test results, the result closest to 48 months was chosen for analysis.

### Cranial ultrasound

At our institution, all infants admitted to the neonatal intensive care unit underwent initial cranial ultrasound within three days of birth. All examinations were performed by board-certified pediatric radiologists using one of the following ultrasound systems: LOGIQ E9 (GE Healthcare, Waukesha, WI, USA) or EPIQ Elite (Philips Medical Systems, Bothell, WA, USA). Coronal and sagittal images were obtained through the anterior fontanelle using either a small convex (3–10 MHz) or linear transducer (6–15 MHz). Transverse images of the posterior fossa were acquired through the mastoid fontanelle.

### Magnetic resonance imaging parameters

All MRIs were acquired using a 3.0T system (Signa Premier; GE Healthcare, Waukesha, WI, USA). The protocol included sagittal 3D magnetization-prepared rapid gradient-echo sequences with the following parameters: repetition time, 2400 ms; echo time, 2.4 ms; inversion time, 1200 ms; flip angle, 12°; field of view, 180 × 180 mm²; and voxel size, 1 × 1 × 1 mm³. These images were reformatted into axial and coronal planes with a slice thickness of 1 mm. The MRI protocol also included axial T1- and T2-weighted turbo spin-echo sequences, susceptibility-weighted imaging, and diffusion-weighted imaging (DWI). DWI was acquired with b-values of 0 and 1000 s/mm^2^, and apparent diffusion coefficient maps were subsequently generated.

### Deep learning-based brain segmentation algorithm

**Fig. 2:**
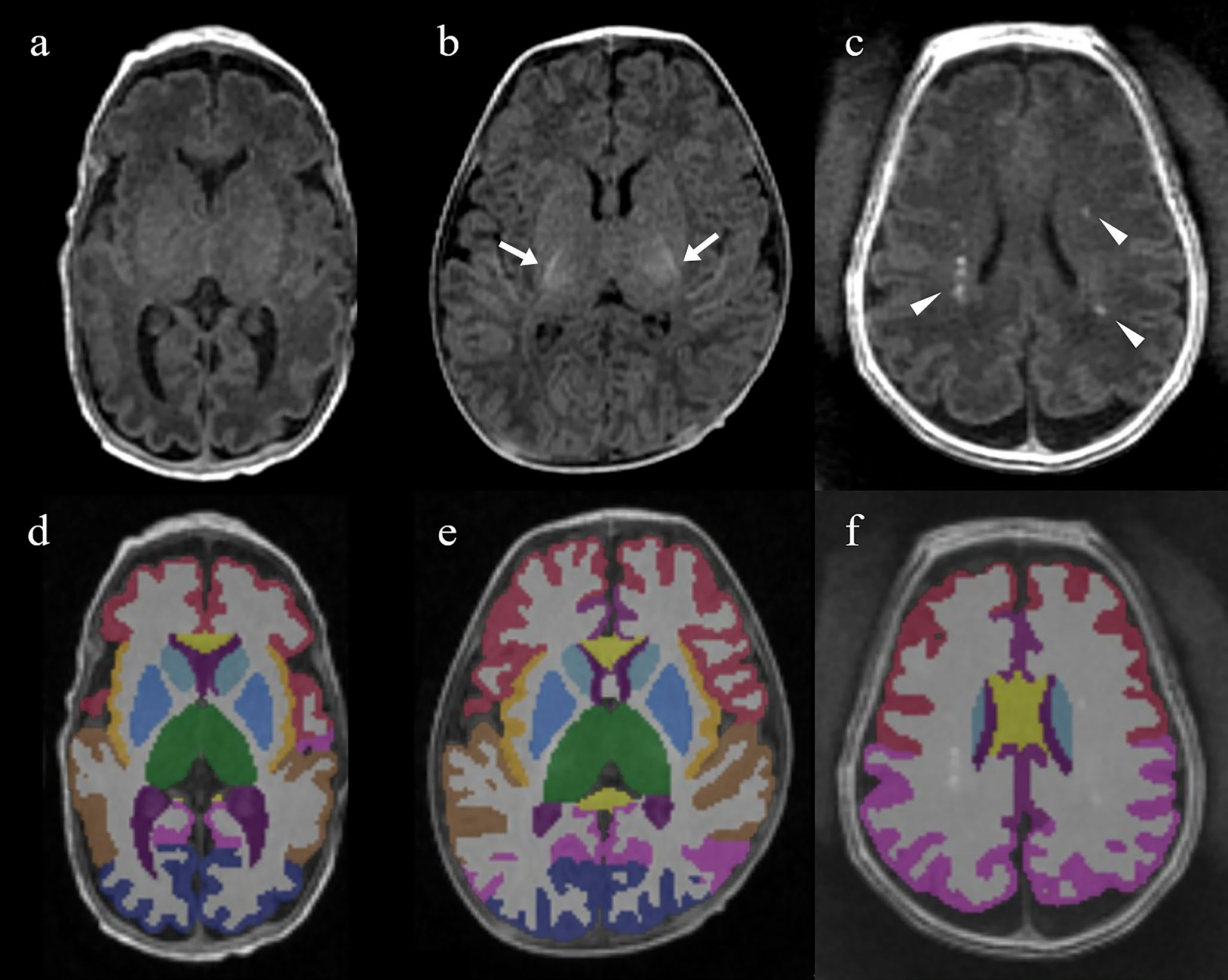
Representative T1-weighted brain magnetic resonance imaging (**a**–**c**) and corresponding segmentation images (**d**–**f**) across different myelination stages and in punctate white matter lesion (PWML). (**a**, **d**) Male infant born at 26 weeks’ gestation and imaged at postmenstrual age (PMA) 35 weeks. (**b**, **e**) Male infant born at 35 weeks’ gestation and imaged at PMA 45 weeks, showing myelination in the posterior limb of the internal capsule (arrows). (**c**, **f**) Female infant born at 29 weeks’ gestation and imaged at PMA 36 weeks, showing bilateral PWML in the periventricular white matter (arrowheads). Myelinated white matter and PWML were correctly segmented as white matter. In the segmentation image, red, pink, brown, blue, purple, yellow, violet, sky blue, green and gray correspond to the frontal lobe, parietal lobe, temporal lobe, occipital lobe, cingulate cortex, insula, lateral ventricle, caudate, thalamus, and white matter, respectively. PWML, punctate white matter lesion; PMA, postmenstrual age

Automatic brain segmentation was performed using commercially available software (Neurophet Aqua; Neurophet Inc., Seoul, Republic of Korea), which is based on the U-Net architecture [24]. Figure 2 shows representative segmentation examples across different myelination stages (PMA 35 and 45 weeks) and in cases with PWML. The development process of this software involved preprocessing steps, including intensity rescaling to a range of 0–1, right-anterior-superior reorientation, and voxel resampling to a size of 1 × 1 × 1 mm³. Data augmentation techniques included gamma correction, Gaussian filtering, simulation of ghosting artifacts, bias field distortion, affine transformation, and elastic deformation. The training data consisted of 206 pairs of T1- and T2-weighted images. Each image was randomly cropped into 64 × 64 × 64 patches, with half of the training patches derived from real MRI scans and the other half from synthetic images generated using a Gaussian mixture modeling–based method. The model demonstrated a Dice similarity coefficient of 0.850 ± 0.032 and an average symmetric surface distance of 0.400 ± 0.070 mm on T1-weighted images. The mean processing time per MRI scan was 7 ± 4 s. Further details on model development are described in the previous study [18].

The algorithm segmented 30 brain regions commonly used in neonatal studies, which were derived from infant FreeSurfer and the dHCP pipeline [25, 26]. Volume for each segmented region was calculated as the sum of its segmented voxels on the 3D T1-weighted sequence. Given the 1 × 1 × 1 mm^3^ voxel size, this directly yielded the volume in cubic millimeters. Supratentorial and white matter volumes were subsequently analyzed.

### Factors associated with neurodevelopmental outcomes

The supratentorial volume–PMA slope, derived from linear regression analysis, was compared among the control, GMH, and PWML groups to assess the association between mild brain injury and brain volume. Standardized residuals were used to assess deviations in brain volume from the expected values based on sex and PMA. A reference standard was established by performing sex-specific linear regressions of supratentorial and white matter volumes against PMA in the control group. The standardized residuals of supratentorial and white matter volume were calculated for each patient in the control, GMH, and PWML groups using the following equation.

$$\begin{array}{lc}\mathrm{Standardized}\;\mathrm{residual}=\frac{\mathrm{Calculated}\;\mathrm{volume}-\mathrm{reference}\;\mathrm{volume}}{\mathrm{Standard}\;\mathrm{deviation}\;\mathrm{of}\;\mathrm{residuals}}\end{array}$$

$$\begin{array}{lc}\mathrm{Standard\;deviation\;of\;residuals}=\\\sqrt{\frac{\sum\:{(\mathrm{Calculated}\;\mathrm{volume}-\mathrm{reference}\;\mathrm{volume})}^2}{n-2}}\end{array}$$

To investigate factors associated with NDI, logistic regression was performed using the presence of NDI as the dependent variable and gestational age at birth, birth weight, standardized residuals, presence of low-grade GM-IVH, and the interaction term between standardized residuals and presence of low-grade GM-IVH as independent variables. Separate analyses were conducted using standardized residuals from supratentorial and white matter volumes. The interaction term was included to assess whether the effect of brain volume on NDI was modified by the presence of low-grade GM-IVH. Standardized residuals were compared between the NDI and non-NDI groups within both the control and GMH groups.

### Statistical analysis

A one-way analysis of variance with Tukey’s post hoc test was used to assess differences in cohort characteristics between groups. A two-proportion z-test was used to evaluate differences in NDI incidence between groups. Pearson correlation coefficients between PMA and supratentorial volume were calculated for each group, and a z-test was used to compare volume growth between groups. The variance inflation factor (VIF) was calculated to assess multicollinearity among independent variables. A VIF < 5 indicated low, 5–10 moderate, and ≥ 10 severe multicollinearity, warranting correction [27]. A two-way analysis of variance with Tukey’s post hoc test was used to assess differences in standardized residuals between NDI subgroups within the control and GMH groups. A *P* value < 0.05 was considered statistically significant. All statistical analyses were performed using Python (v.3.12.2; Python Software Foundation, Wilmington, DE, USA) with the following libraries: Matplotlib (v.3.9.2), Pandas (v.2.2.3), Seaborn (v.0.11.2), SciPy (v.1.14.1), and NumPy (v.2.1.2).

## Results

### Demographics of the study cohort

The demographics of the study cohort are presented in Table 1. A total of 171 preterm infants were enrolled (89 girls; gestational age at birth, 28.32 ± 2.76 weeks; birth weight, 1049.38 ± 445.29 g; PMA at MRI acquisition, 37.81 ± 2.36 weeks). The numbers of patients in the control, GMH, and PWML groups were 119, 41, and 11, respectively. No significant differences in demographics were observed among the groups. The Bayley-III test was conducted on 47 of 119 (40%) patients in the control group, 25 of 41 (61%) in the GMH group, and 3 of 11 (27%) in the PWML group. NDI was observed in nine patients each from the control and GMH groups, and none was observed in the PWML group. Although the incidence of NDI was higher in the GMH group (9 of 25, 36%) than in the control group (9 of 47, 19%), this difference was not statistically significant (*P* = 0.08).

**Table 1:** Demographics of the study cohort

Variable	Control group (*n* = 119)	GMH group (*n* = 41)	PWML group (*n* = 11)	*P* value
Sex (male: female)	57:62	20:21	5:6	0.98
Gestational age at birth (weeks)	28.42 ± 2.60	27.46 ± 2.27	29.67 ± 3.32	0.10
Birth weight (g)	1046.97 ± 452.38	965.51 ± 329.03	1088.09 ± 606.40	0.12
Postmenstrual age at MRI acquisition (weeks)	37.59 ± 2.19	38.34 ± 2.46	38.18 ± 3.42	0.19

Values indicate the mean ± standard deviation. *P* values were calculated using one-way analysis of variance among the control, GMH, and PWML groups. GMH, germinal matrix hemorrhage; PWML, punctate white matter lesion; MRI, magnetic resonance imaging

### Supratentorial volume distribution in mild brain injury

**Fig. 3:**
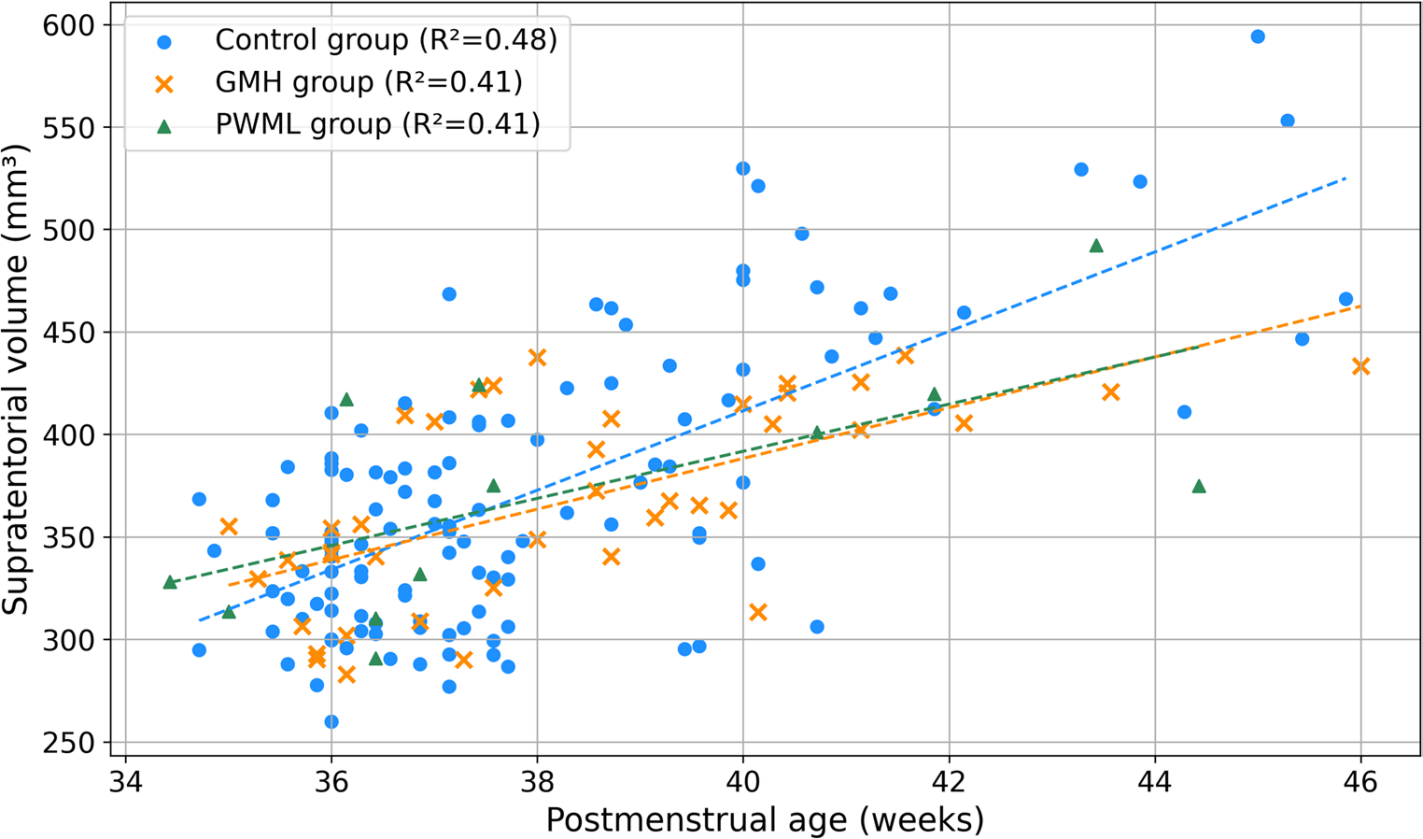
Supratentorial volume distribution in the control, germinal matrix hemorrhage (GMH), and punctate white matter lesion (PWML) groups. Linear regression analysis of supratentorial volume by postmenstrual age in the control, GMH, and PWML groups. The slope was 19.37 cm³/week in the control group, 12.37 cm³/week in the GMH group, and 11.49 cm³/week in the PWML group (control vs. GMH, *P* = 0.02; control vs. PWML, *P* = 0.04; GMH vs. PWML, *P* = 0.70). GMH, germinal matrix hemorrhage; PWML, punctate white matter lesion

The mean ± standard deviation of supratentorial volume was 369.37 ± 66.80 cm³ in the control group, 367.75 ± 47.77 cm³ in the GMH group, and 373.29 ± 60.09 cm³ in the PWML group, with no significant difference between the groups (*P* = 0.96). The supratentorial volume–PMA slope was 19.37 cm³/week (R² = 0.48; 95% confidence interval [CI], 16.82–21.92) in the control group, 12.37 cm³/week (R² = 0.41; 95% CI, 7.67–16.06) in the GMH group, and 11.49 cm³/week (R² = 0.41; 95% CI, 4.94–16.70) in the PWML group (Fig. 3). Preterm infants with low-grade GM-IVH and PWML had significantly lower slopes compared to the control group (control vs. GMH group, *P* = 0.02; control vs. PWML group, *P* = 0.04; GMH vs. PWML group, *P* = 0.70).

### Factors associated with neurodevelopmental outcomes

**Fig. 4:**
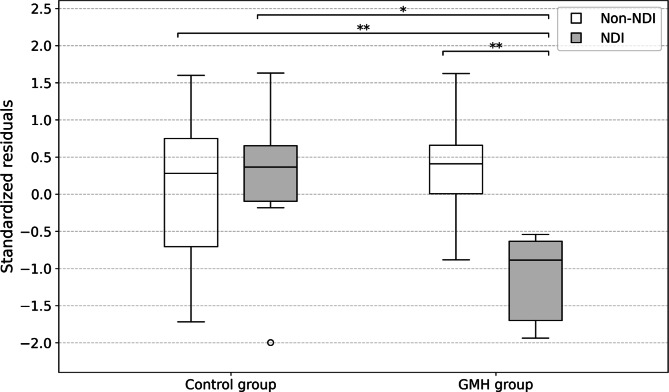
Supratentorial volume according to neurodevelopmental impairment (NDI) status in control and germinal matrix hemorrhage (GMH) groups. The asterisks between two box plots indicate a statistically significant difference (*P < 0.05, **P < 0.001). NDI, neurodevelopmental impairment; GMH, germinal matrix hemorrhage

**Fig. 5:**
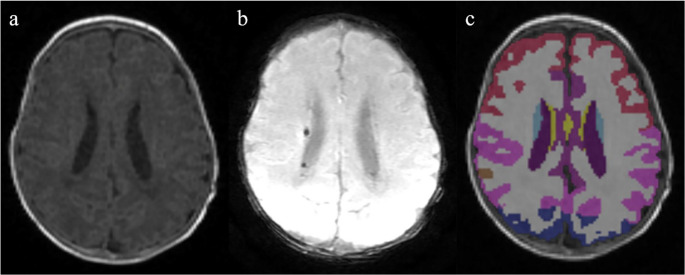
Brain magnetic resonance imaging of a female neonate born at 24 weeks of gestation with images acquired at 40 weeks’ postmenstrual age. (**a**) Three-dimensional T1-weighted image; (**b**) susceptibility-weighted image; and (**c**) segmentation image. Low-grade germinal matrix-intraventricular hemorrhage was observed on brain ultrasonography and susceptibility-weighted imaging. Volumetric analysis showed a volume reduction of −1.93 standard deviations. The Bayley-III test at 48 months confirmed neurodevelopmental impairment, with scores of 65, 67, and 67 in the cognitive, language, and motor domains, respectively. In the segmentation image, red, pink, brown, blue, purple, yellow, violet, sky blue, and gray correspond to the frontal lobe, parietal lobe, temporal lobe, occipital lobe, cingulate cortex, insula, lateral ventricle, caudate, and white matter, respectively.

Standardized residuals of supratentorial volume are shown in box plots according to the presence of NDI in the control and GMH groups (Fig. 4). In the control group, there was no significant difference in the mean standardized residual of supratentorial volume between the NDI and non-NDI subgroups (0.14 ± 1.21 vs. 0.11 ± 0.95, *P* = 0.95). Conversely, in the GMH group, the mean standardized residual of supratentorial volume was significantly lower in the NDI subgroup than in the non-NDI subgroup (−1.12 ± 0.58 vs. 0.35 ± 0.69, *P* < 0.001). A representative case in the NDI subgroup is shown in Fig. 5.

Logistic regression analysis yielded odds ratios of 0.65 for gestational age at birth (per 1-week increase; 95% confidence interval [CI], 0.30–1.39; *P* = 0.26), 0.96 for birth weight (per 10-g increase; 95% CI, 0.86–1.05; *P* = 0.56), 0.68 for standardized residuals (per 1-unit increase; 95% CI, 0.18–1.27; *P* = 0.58), 1.42 for the presence of low-grade GM-IVH (95% CI, 0.68–2.37; *P* = 0.13), and 1.50 for the interaction between standardized residuals and low-grade GM-IVH (95% CI, 1.23–1.78; *P* = 0.03). VIF values were 3.72 for gestational age, 5.67 for birth weight, 2.17 for standardized residuals, 1.03 for presence of low-grade GM-IVH, and 1.56 for the interaction term, indicating low multicollinearity for all variables except birth weight, which showed moderate multicollinearity. Overall, only the interaction term reached statistical significance, suggesting that the presence of low-grade GM-IVH modifies the association between standardized residuals and NDI.

**Fig. 6:**
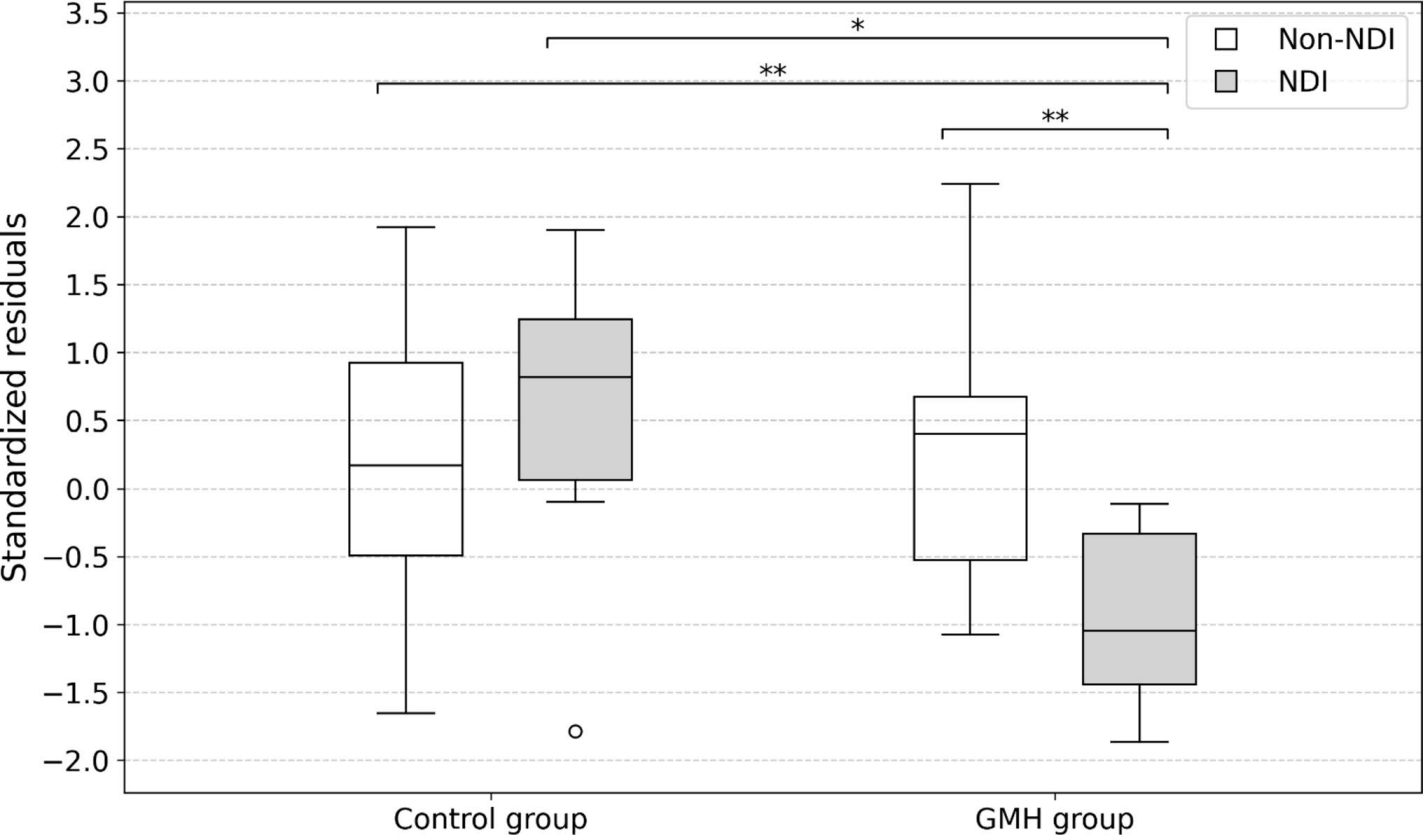
White matter volume according to neurodevelopmental impairment (NDI) status in control and germinal matrix hemorrhage (GMH) groups. The asterisks between two box plots indicate a statistically significant difference (*P <0.05, **P <0.001). NDI, neurodevelopmental impairment; GMH, germinal matrix hemorrhage

Similarly, for white matter volume, standardized residuals are presented as box plots (Fig. 6). In the control group, there was no significant difference in the mean standardized residual of white matter volume between the NDI and non-NDI subgroups (0.49 ± 1.31 vs. 0.17 ± 0.94, *P* = 0.57). Conversely, in the GMH group, the mean standardized residual of white matter volume was significantly lower in the NDI subgroup than in the non-NDI subgroup (− 0.95 ± 0.68 vs. 0.29 ± 0.92, *P* < 0.001).

Logistic regression analysis yielded odds ratios of 0.83 for gestational age at birth (per 1-week increase; 95% confidence interval [CI], 0.50–1.16; *P* = 0.34), 0.95 for birth weight (per 10-g increase; 95% CI, 0.85–1.05; *P* = 0.49), 0.54 for standardized residuals (per 1-unit increase; 95% CI, 0.15–1.97; *P* = 0.35), 2.77 for the presence of low-grade GM-IVH (95% CI, 0.43–17.81; *P* = 0.28), and 1.33 for the interaction between standardized residuals and low-grade GM-IVH (95% CI, 0.97–1.70; *P* = 0.07). VIF values were 3.47 for gestational age, 4.04 for birth weight, 2.14 for standardized residuals, 1.03 for presence of low-grade GM-IVH, and 1.74 for the interaction term, indicating low multicollinearity for all variables. No variables reached statistical significance; however, the interaction term yielded the lowest *P* value among the variables (*P* = 0.07).

## Discussion

In this study, supratentorial and white matter volumes were lower in the GMH group with NDI than in both the control group and the GMH group without NDI, suggesting that reduced brain volume in infants with low-grade GM-IVH may indicate a pathological outcome.

Approximately 11% of neonates are born preterm, before 37 weeks of gestation, with a reported prevalence of at least 7% across countries, regardless of their level of development [28]. A substantial proportion of preterm neonates develop GM-IVH, and the risk inversely correlated with gestational age at birth [3, 4]. This vulnerability is attributed to the highly vascular and structurally fragile nature of the immature germinal matrix. The prognosis of GM-IVH is clinically important, particularly regarding the impact of low-grade GM-IVH (Papile grades 1 and 2) on neurodevelopment, as it affects a larger proportion of preterm infants than high-grade GM-IVH. Previous studies have shown that high-grade GM-IVH increases the risk of cognitive, language, and motor impairments, as well as cerebral palsy and sensory deficits [6–8]. Moreover, iron in the form of hemosiderin and ferritin accumulates in the periventricular white matter after high-grade GM-IVH, even in regions that appear normal on conventional MRI, indicating ongoing iron-mediated white matter injury [29].

However, the prognosis of low-grade GM-IVH remains unclear, particularly regarding its potential to increase neurodevelopmental risk. Meta-analyses have reported increased odds of NDI in preterm infants with low-grade GM-IVH, with an adjusted odds ratio of 1.39 (95% CI, 1.09–1.77) as reported by Mukerji et al. [30] and an unadjusted odds ratio of 1.20 (95% CI, 1.08–1.34) from Zhou et al. [9]. However, other studies found no significant difference in the presence of NDI between infants with low-grade GM-IVH and those without hemorrhage [6–8, 31]. Although the impact of low-grade GM–IVH is generally considered less severe than that of high-grade lesions, a previous study suggested that it may contribute to brain injury independent of ventricular dilatation and be associated with adverse neurodevelopmental outcomes [32]. Regional cerebral hypoperfusion in the posterior cortex and deep gray matter has been reported in preterm neonates with low-grade GM–IVH [33], while moderate and late preterm infants may show region-specific alterations in structural and functional brain connectivity, suggesting potential implications for neurodevelopmental outcomes even in low-grade GM–IVH [34].

In addition, accompanying factors associated with low-grade GM–IVH that may contribute to the risk of neurodevelopmental impairment have not been thoroughly explored in previous studies. Brain volume may be one such factor, given its known association with neurodevelopmental outcomes in preterm infants [31, 35]. One study reported reduced medullary volume in low-grade GM-IVH, with no significant differences in other brain regions [31]. However, the volumetric analysis was based on two-dimensional T1-weighted images and a relatively small cohort (*n* = 25).

According to our results, the absence of a volume difference between the NDI and non-NDI subgroups within the control group indicates that brain volume alone cannot be considered a determinant of neurodevelopmental outcome. However, in the GMH group, a significant difference in brain volume was observed between the NDI and non-NDI subgroups. In addition, logistic regression analysis demonstrated a significant interaction term, indicating that the presence of low grade GM-IVH modifies the effect of brain volume on the risk of NDI. Although previous studies have consistently reported that lower gestational age at birth and lower birth weight are associated with an increased risk of neurodevelopmental delay [36–38], brain volume did not provide additional predictive information for neurodevelopmental outcomes in this control population. Reduced cerebral volume in the control group is likely part of a normal developmental trajectory rather than indicative of a structural abnormality, with neurodevelopment in these infants progressing as expected [39, 40]. Given that the germinal matrix is composed of progenitor cells that generate neurons and glial cells and is essential for angiogenesis, a reduction in volume with low grade GM-IVH may represent a pathological outcome resulting from injury [32].

Previous studies have reported that the number and extent of PWML are associated with adverse motor outcomes, including cerebral palsy and abnormal muscle tone, likely due to involvement of motor pathways such as the corticospinal tract [13, 41, 42]. Although many studies have shown no association between PWML and language or cognitive outcomes [13, 42, 43], one study reported a correlation between the number of PWML and cognitive disability [41]. Our study aimed to evaluate the association between PWML with volume reduction and the prevalence of NDI; however, no patients with PWML were diagnosed with NDI in our cohort, making such analysis infeasible and warranting further investigation. Patients with PWML appeared to have reduced brain volume growth at term-equivalent age, possibly related to loss of axonal integrity.

Reliable brain volumetric analysis is essential to support our findings. In infants, brain segmentation is more challenging than in adults because of incomplete myelination, which reduces the signal intensity contrast between white and gray matter, as well as fewer brain sulci and gyri and differences in water content. Recently, U-Net–based infant brain segmentation has demonstrated good performance compared with manual annotation, with Dice similarity coefficients ranging from 0.82 to 0.95 [18–20]. Despite consistent performance degradation in external validation due to scanner and protocol differences, these models maintained high accuracy. The specific model used in this study achieved a Dice similarity coefficient of 0.850 ± 0.032 and an average symmetric surface distance of 0.400 ± 0.070 mm on external T1-weighted image testing [18]. While the model was trained and validated on normal infants, our analysis focused on mild brain injury, where low-grade GM-IVH is typically not visible on T1-weighted images and PWML is expected to minimally impact brain volume. Therefore, the segmentation is considered reliable when combined with the high-resolution imaging in our study. The infant FreeSurfer pipeline is a widely used brain segmentation algorithm that demonstrates moderate performance (Dice similarity coefficient 0.5–0.7 in infants for the cerebral cortex and white matter), but its long processing time limits its clinical applicability [44]. The processing time of the model used in our study was within a few minutes on a CPU-based workstation commonly available in clinical settings. This supports its feasibility for real-world application, particularly in identifying preterm infants with GM-IVH and reduced brain volume who may be at increased risk of adverse neurodevelopmental outcomes.

This study has several limitations. First, since the study population comprised preterm infants admitted to a tertiary hospital neonatal intensive care unit, the control group may not accurately represent the typical developmental trajectory of the general population. Second, the sample size was relatively small. Although the Bayley-III test is recommended for preterm infants in the toddler stage at our institution, it was not performed in all cases, potentially introducing selection bias. Therefore, a longitudinal study with a larger cohort and extended follow-up is required to evaluate the long-term effects of low-grade GM-IVH. Third, although brain volume segmentation performance is likely to differ across age groups within the 34–46 weeks PMA range, no age-stratified analysis was conducted. However, given that segmentation in neonates is more challenging than in older infants, the model’s good performance in neonates supports the acceptability of the volumetric analysis within this age range. Fourth, Although the segmentation model was trained on normal neonates, it was applied to infants with mild brain injury in this study. However, as low grade GM-IVH is not clearly distinguishable on T1-weighted images and PWML was visually confirmed to be segmented as white matter, the potential impact on segmentation accuracy was considered minimal. Fifth, the physiological mechanisms underlying the association between reduced brain volume in low-grade GM-IVH and increased risk of NDI were not thoroughly evaluated, and further research is needed to clarify them.

## Conclusion

Using a deep learning-based brain segmentation model, we found that the association between reduced supratentorial or white matter volume and an increased risk of NDI was primarily observed in preterm infants with low-grade GM-IVH. This association was not evident in infants without GM-IVH. Our findings suggest that decreased brain volume may serve as an important prognostic indicator for adverse neurodevelopmental outcomes in this group. Further large-scale, long-term studies are warranted to validate these results.

## Data Availability

Data not provided in the manuscript are available from the corresponding author upon reasonable request.
